# A178 DISEASE COURSE AND MANAGEMENT OF CHILDREN WITH VERY EARLY ONSET INFLAMMATORY BOWEL DISEASE

**DOI:** 10.1093/jcag/gwae059.178

**Published:** 2025-02-10

**Authors:** S B Kishore, A Sethuraman, H Binomar, K Jacobson

**Affiliations:** Griffith University School of Medicine and Dentistry, Gold Coast, Queensland, Australia; BC Children’s Hospital, Vancouver, BC, Canada; King Faisal Specialist Hospital and Research Centre, Riyadh, Riyadh, Saudi Arabia; The University of British Columbia Faculty of Medicine, Vancouver, BC, Canada

## Abstract

**Background:**

Very early-onset inflammatory bowel disease (VEO-IBD), defined as IBD diagnosed before 6 years of age, has a distinct phenotype with predominantly colonic involvement. However, there are limited studies describing the long-term outcome of children with VEO-IBD.

**Aims:**

Our primary aim was to determine the long-term outcome in children with VEO-IBD and corticosteroid free clinical remission (CFCR) at one year post diagnosis and at last follow up. The secondary aim was to determine clinical response (CR) and biochemical remission (BR) at the same time points.

**Methods:**

A retrospective cross-sectional chart review was conducted for all children with VEOIBD diagnosed between 1^st^ January 2013 to 31^st^ December 2022 with a minimum follow up of one year at British Columbia Children’s Hospital (BCCH). Patients were identified from the BCCH pediatric IBD database. Demographic and relevant clinical data including IBD medications and response were collected. Data points at 3 months, 6months, 1 year and then annually until 10 years from diagnosis were collected. Corticosteroid free clinical remission (CFCR) was defined as being off steroids for ≥ 3 months at yearly follow up. Biochemical remission (BR) was defined as fecal calprotectin <250ug/g. Clinical response (CR) was defined as a wPCDAI >17.5-point change or a PUCAI of a 20-point change from baseline.

**Results:**

77 patients were identified, with 70 patients reviewed thus far. The mean age at diagnosis was 45.6 (±16.4 months). The median follow-up time was 60 months (interquartile range 36 to 84 months). Of the 38 (54%) patients on biologic therapy, the most common 1st, 2^nd^, 3^rd^ and 4^th^ line biologics were anti-TNF (25/26), vedolizumab (4/7), ustekinumab (4/4) and vedolizumab (1/1) respectively.

**Conclusions:**

Children with VEOIBD who have milder disease and do not require biologic therapy achieved better rates of CFCR at 1 year follow up (83.3% compared to 52.7%) but comparable rates at last follow up (78.1% to 81.6%) to those with moderate to severe disease requiring biologic therapy.

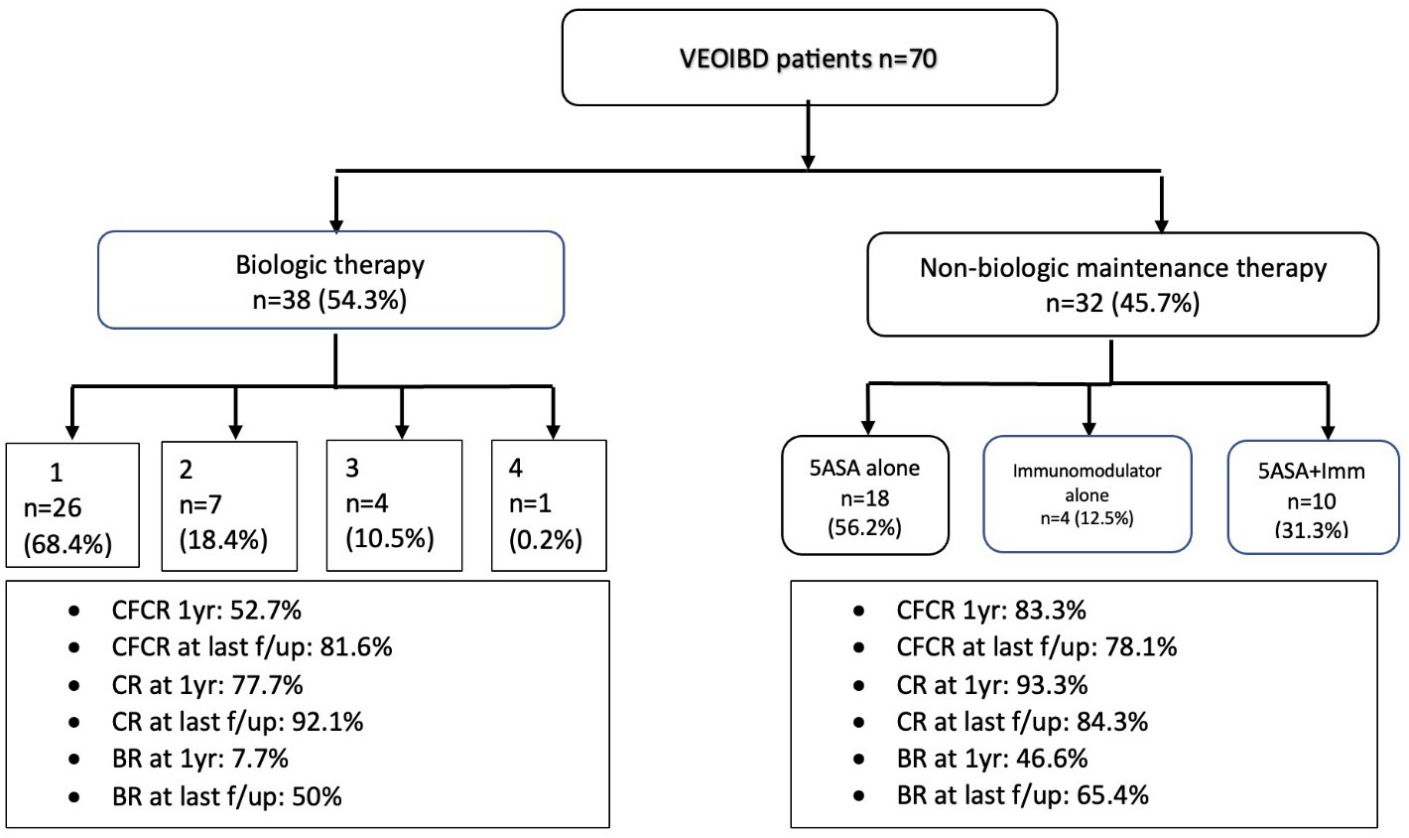

Primary and secondary outcomes in VEOIBD patients

**Funding Agencies:**

None

